# Chaotic fractals: Why chaos is the dynamic of carcinogenesis

**DOI:** 10.1002/cai2.63

**Published:** 2023-03-30

**Authors:** Mesut Tez

**Affiliations:** ^1^ Department of Surgery, Ankara City Hospital University of Health Sciences Ankara Turkey

**Abbreviations**
AFM: atomic force microscopy
CAS: complex adaptive system
CT: computed tomography
FD: fractal dimension

## 1 INTRODUCTION

We can see the fractals in our environment every day (trees, snowflakes, broccoli, etc.). Even the shapes of the DNA helix and anatomical structures are fractal, for example, the branching of blood vessels, bronchi, and cell membranes [1]. Unlike euclidean geometry, fractal geometry reveals how an object with irregularities in many dimensions can be identified by examining how the number of features in one dimension relates to the number of similarly shaped features in other dimensions [2]. Mandelbrot used fractal geometry to describe such irregular shapes and demonstrated that this geometry was an appropriate mathematical language for describing chaotic systems [1]. In fractal geometry, the fractal dimension (FD) is a statistical quantity that gives an indication of how completely a fractal appears to fill space, as one zooms down to finer and finer scales. The FD provides a measure of the complexity of a structure. Increased FD is an indicator of chaos [3].

## 2 WHAT IS THE RELATION OF THESE UNUSUAL AND BEAUTIFUL GEOMETRICAL STRUCTURES TO CANCER?

A complex adaptive system (CAS) is a type of system that is composed of many interacting components, called agents, which can adapt and change their behavior based on their interactions with the environment and with other agents. CAS are characterized by their ability to self‐organize and evolve over time, often resulting in emergent properties and behaviors that cannot be predicted from the properties of the individual agents alone. Examples of CAS include ecosystems, economies, social networks, and the human brain. It is also worth noting that a CAS can have both chaotic and regular behavior depending on the circumstances and the complexity of the system. Stem cells can also be considered CAS because they possess many of the characteristics that define CAS. Stem cells have the ability to self‐renew, differentiate into multiple cell types, and respond to signals from their environment [4]. Some studies suggest an important role of the feedback loop between cancer cells and the microenvironment. Also, putting cells into an “inappropriate” microenvironmental context can otherwise trigger pathological issues, and even neoplastic transformation [5]. Cancer has previously been demonstrated to be a chaotic behavior of the stem cell [6].

## 3 CHAOTIC FDS IN CANCER TISSUES/CELLS

The FD of chromatin has been demonstrated to increase during carcinogenesis and tumor growth in diffuse large B‐cell lymphoma, chronic lymphocytic leukemia, oropharyngeal carcinoma, and hepatocarcinoma compared to equivalent normal tissue. A research study of over 3000 cancer specimens revealed the prevalence of fractal chromatin structure in neoplasias, as well as the importance of this arrangement in the creation of chromosomal abnormalities [7]. Fractal analysis of the cell surface is a rather sensitive method that has been recently introduced to characterize cell progression toward cancer. Analysis of FD of cell surface imaged with atomic force microscopy (AFM) showed strong segregation between normal and malignant human cervical epithelial cells [8]. There is a growing literature that shows FD to be a useful measure of the pathologies of the tumoral vascular architecture, and tumor/parenchymal border [3, 9].

## 4 CHAOTIC FDS IN THE TISSUE LEVEL

Entropy‐based fractal image modeling has been used to increase the diagnostic accuracy of mammographic detection of breast tumors. Mammographic density correlates with the FD of breast cancer and eventually with a higher growth rate. Breast masses with higher FD show increased aggressiveness and poor prognosis [5]. The FD of the lung tumor on contrast‐enhanced computed tomography (CT) images demonstrated that the use of FD as a predictive indicator of therapeutic response or progression is warranted [10]. Similarly, the FD of liver mass in contrast‐enhanced CT is a useful prognostic biomarker for hepatocellular carcinoma patients treated with sunitinib [11]. The FD analysis of thyroid ultrasound images is used for the prediction and early detection of thyroid malignancy [12]. Moreover, fractal geometry is applied in many radiographic analyses.

## 5 CONCLUSION

At least, the application of chaos theory opens up the opportunity for novel dynamic access to carcinogenesis. Of course, the application of chaos theory cannot solve all problems, but such an interdisciplinary approach may increase the understanding of carcinogenesis.

## AUTHOR CONTRIBUTION

**Mesut Tez**: Writing—review and editing (equal).

## CONFLICT OF INTEREST STATEMENT

The author declares no conflict of interest.

## ETHICS STATEMENT

Not applicable.

## INFORMED CONSENT

Not applicable.

## Data Availability

If no data are available, there is no data for this review.
